# HIV en mujeres mayores de 50 años. Análisis de datos administrativos en departamentos del Pacífico colombiano (2014-2018)

**DOI:** 10.7705/biomedica.5697

**Published:** 2021-10-15

**Authors:** Juanita Camacho, Diana Moscote, Yoseth J. Ariza

**Affiliations:** 1 Programa de Medicina, Universidad Icesi, Cali, Colombia Universidad Icesi Universidad Icesi Cali Colombia; 2 Departamento de Salud Pública y Medicina Comunitaria, Universidad Icesi, Cali, Colombia Universidad Icesi Universidad Icesi Cali Colombia

**Keywords:** HIV, mujeres, anciano, epidemiología, prevalencia, medio social, prevención de enfermedades, HIV, women, aged, epidemiology, prevalence, social environment, disease prevention

## Abstract

**Introducción.:**

El HIV es un problema de salud pública relevante en el Pacífico colombiano debido a la gran problemática social de la región y la exposición de las mujeres a más factores de riesgo.

**Objetivo.:**

Describir las diferencias por sexo y grupos de edad con respecto al código CIE-10 de HIV más frecuente en los cuatro departamentos de la Región Pacífica colombiana durante el periodo de 2014 a 2018.

**Materiales y métodos.:**

Se hizo un estudio observacional descriptivo, retrospectivo y con unidades de análisis ecológicas (departamentos y municipios) obtenidas por agregación. La fuente de datos fue el SISPRO, una plataforma para el registro obligatorio de las prestaciones de servicios de salud en el país. Para cada grupo de edad y ente territorial, se estimaron las proporciones del código para mujeres y para hombres, y se calcularon las diferencias absolutas (la proporción femenina menos la proporción masculina).

**Resultados.:**

A nivel regional, en 9 de 22 grupos se observó el predominio de mujeres, con la mayor diferencia en el grupo de 25 a 29 años. Cinco de los nueve grupos de edad con mayor reporte en mujeres correspondían a mayores de 50 años. La mayor diferencia se encontró en el grupo de 50 a 54 años en Chocó y, la menor, en el grupo de 90 a 94 años en Nariño. En el análisis se identificaron dos patrones: antes de los 50 años, con predominio de los hombres, y después de los 50 años, con predominio de las mujeres.

**Conclusiones.:**

El análisis secundario de una base de datos de recolección rutinaria es útil. Dado que los datos evidenciaron una prevalencia de los adultos mayores, especialmente mujeres, es importante que se considere incluir esta población en las estrategias de promoción y prevención del programa de HIV.

## Abstract (en)

**Introduction:**

HIV is a public health problem that is relevant in the Colombian Pacific due to major social problems in the region where women are exposed to more risk factors.

**Objective:**

To describe the differences by gender and age of the most frequent ICD-10 code related to HIV in four departments of the Colombian Pacific region during the period 2014 to 2018.

**Materials and methods:**

We conducted a descriptive, retrospective observational study with ecological units of analysis (departments and municipalities) obtained by aggregation. The data source was SISPRO, a platform for the mandatory registration of health services in the country. For each age group and territorial entity, we estimated the proportions of each code for women and men, as well as the absolute differences (the female proportion minus the male proportion).

**Results:**

At the regional level, there was a predominance of women in 9 of 22 groups evaluated. The largest absolute difference was in the 25-29 age group. Five of the nine age groups with a predominance of women represented ages over 50 years. The greatest difference was found in the 50-54 age group in Chocó, and the smallest difference was found in the 90-94 age group in Nariño. The analysis identified the following two patterns: before the age of 50 with a predominance of men and over the age of 50 with a predominance of women.

**Conclusions:**

The secondary analysis of databases for routine information collection is useful. Given that the data showed a prevalence of older adults, especially women, it is important to consider including this population in the promotion and prevention strategies of the HIV program.

El HIV se considera un problema de salud pública a nivel mundial y nacional debido al gran impacto en la calidad de vida de las personas que lo padecen y sus familias, así como en la economía, ya que se clasifica como una condición de alto costo tanto para la población como para el sistema de salud del país. Se considera que las mujeres son especialmente vulnerables a la infección por HIV; según ONUSIDA, en 2016 de todos los infectados, 17,8 millones (IC95% 15,4-20,3 millones) eran mujeres mayores de 15 años (^1^). Según los *Centers for Disease Control and Prevention* (CDC) de Estados Unidos, el 48 % de la población infectada con HIV en ese país es mayor de 50 años (^2^). En Colombia, el 20 % de la población diagnosticada con HIV corresponde a mujeres mayores de 15 años (^1^). La población femenina colombiana tiene una gran vulnerabilidad ante la infección de HIV debido a múltiples factores como la exclusión, la desigualdad, la marginalidad y, sobre todo, la pobreza, la cual es muy prevalente en el país, especialmente en la región del Pacífico colombiano (^3^,^4^).

En este estudio se propuso analizar la fuente administrativa de datos de mayor cobertura del país para describir las diferencias por sexo y grupos de edad del código CIE-10 relacionado con HIV más frecuente en los cuatro departamentos de la Región Pacífica colombiana durante el periodo de 2014 a 2018.

## Materiales y métodos

### Diseño del estudio y fuente de información

Se hizo un estudio observacional descriptivo, retrospectivo y con unidades de análisis ecológicas obtenidas por agregación de información individual. La fuente de datos fue el Sistema Integrado de Información de la Protección Social (SISPRO), plataforma que ofrece un servicio digital para atender necesidades de información de los ciudadanos y grupos de interés (^5^). La cobertura del sistema es nacional y su uso es de carácter obligatorio para el registro de las prestaciones de servicios de salud. Puesto que el SISPRO da acceso a todos los datos del país, no fue necesario el muestreo. Para las consultas y la descarga de los archivos, se siguieron las recomendaciones presentadas en la capacitación ofrecida por el grupo administrador de la plataforma.

### Procesamiento de los datos

**Figura 1:**
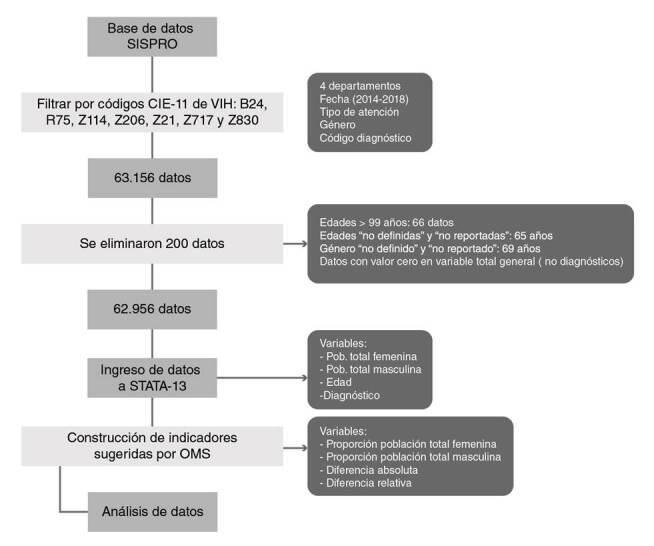
Flujograma del procesamiento de los datos

A partir de la consulta en el SISPRO, se generó la base de datos, la cual incluyó los siguientes campos para cada paciente: identificación, edad, sexo, departamento y municipio de residencia, municipio donde fue proporcionado el servicio, identificación del proveedor, diagnóstico primario, fecha de servicio, tipo de servicio (de procedimiento o farmacológico) y tipo de instalación (hospital, atención ambulatoria, de emergencia). Dado que cada paciente puede usar los servicios de salud más de una vez al año, o recibir más de un servicio en cada visita (como paciente ambulatorio u hospitalizado), los datos se agruparon por identificación para obtener el número de pacientes por año y período. Los códigos de diagnóstico seleccionados correspondieron todos al HIV (^6^): B24, R75, Z114, Z206, Z21, Z717 y Z830. Los grupos de edad se organizaron así: los menores de 25 años se agruparon con base en la propuesta del Ministerio de Salud y Protección Social presentada en la guía de prevención de HIV para jóvenes en contextos de vulnerabilidad en Colombia (^7^), y los mayores de 25 años, en grupos quinquenales, es decir, que en el rango de 0 a 99 años resultaron 22 grupos de edad. En una tabla de Microsoft Excel^®^ se estimaron las proporciones de interés “población total femenina” y “población total masculina”, así: número de mujeres que tuvieron ese diagnóstico en cada grupo de edad sobre el total de mujeres con ese diagnóstico en todas las edades, y de la misma forma se procedió para el sexo masculino. Además, se calculó la diferencia absoluta y la relativa de estas dos poblaciones, siendo la primera la resta entre la proporción femenina y la masculina ya calculadas, y la segunda, la división entre estos dos mismos valores. Dichas operaciones se hicieron con el fin de que los datos estuvieran dispuestos de igual forma que en la propuesta del 2016 hecha por la Organización Mundial de la Salud (OMS) para fortalecer los sistemas nacionales de monitoreo y evaluación del HIV y la salud sexual y reproductiva sensible al género (^8^). En consecuencia, en el presente estudio, el número de mujeres fue el numerador de la división y el minuendo de la diferencia para hacer referencia al grupo poblacional que ha sido sistemáticamente más vulnerable. El número de hombres fue el denominador y el sustraendo para los indicadores descritos. En la figura 1 se ilustra el flujograma del procesamiento de los datos.

### Área de estudio

El análisis se restringió a los cuatro departamentos que constituyen la región geográfica del país con costas en el océano Pacífico (Cauca, Chocó, Nariño y Valle del Cauca) y que, además, comparten características demográficas, históricas y culturales, así como redes de prestación de servicios de salud.

### Análisis estadístico

Las unidades de análisis fueron ecológicas y correspondieron a los departamentos (entes territoriales equivalentes a estados) y los municipios (entes territoriales que conforman los departamentos) de residencia de los pacientes. El periodo de estudio se extendió entre enero del 2014 y diciembre del 2018. Las variables de los municipios y departamentos se obtuvieron por agregación (suma aritmética) de los datos individuales. Para complementar la descripción del nivel departamental, se elaboraron tablas agrupando los municipios según el tipo de distribución poblacional y considerándolos dispersos o concentrados según el punto de corte del 75 % de la población residente en la cabecera municipal con base en lo reportado en las proyecciones censales del Departamento Administrativo Nacional de Estadística (DANE) (^9^). Este análisis estratificado de agregación de municipios de los cuatro departamentos se usó como una estrategia para obtener información sobre los territorios rurales con datos insuficientes en las escalas temporal y espacial usadas habitualmente.

### Consideraciones éticas

El estudio se ajustó a las disposiciones nacionales determinadas por la resolución 8430 de 1993 del Ministerio de Salud de Colombia. El protocolo fue revisado y aprobado por el comité de ética en investigación de la Universidad Icesi.

## Resultados

El diagnóstico que presentó la mayor agregación de población de forma persistente en los municipios y departamentos fue el B24 “Enfermedad por VIH”, por lo cual el análisis se restringió a este código. Los valores obtenidos del cálculo de la diferencia relativa no se tuvieron en cuenta, dado que para muchos de ellos no había datos o no eran calculables porque el denominador (hombres) era cero. Dado lo anterior, los resultados se basaron únicamente en la diferencia absoluta.

En el cuadro 1, se presentan las diferencias absolutas para cada grupo de edad en los cuatro departamentos. Se observó que las mujeres predominaron en 9 de los 22 grupos de edad. La mayor diferencia se encontró en el grupo de 25 a 29 años (^1^,^4^) y, la menor, en el de 20 a 24 años (0,05). Cinco de los nueve grupos de edad con mayor reporte en mujeres correspondieron a mayores de 50 años.

**Figura 2:**
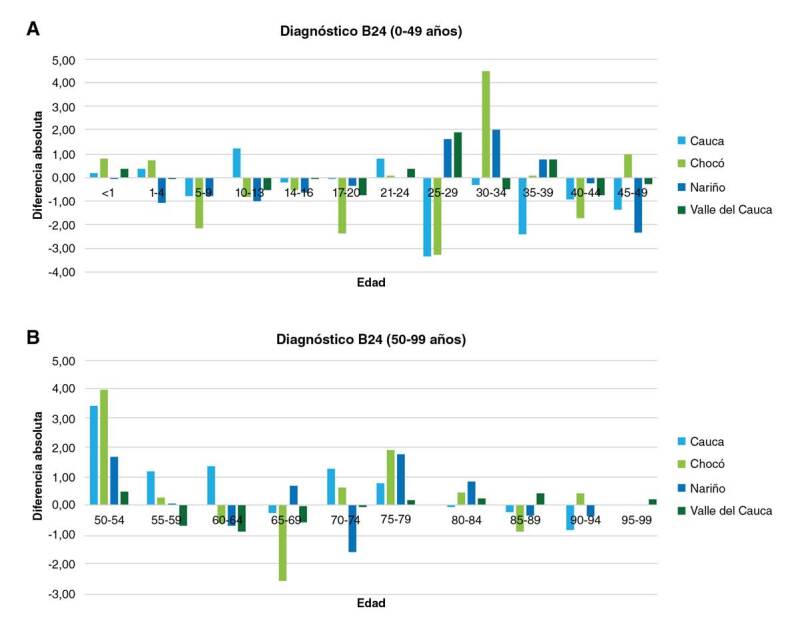
A. Diferencia absoluta frente al diagnóstico B24 por departamentos en menores de 50 años. B. Diferencia absoluta frente al diagnóstico B24 por departamentos en mayores de 50 años

En la parte A de la figura 2 se muestra el comportamiento de las diferencias en los cuatro departamentos para los grupos de edad por debajo de los 50 años y, en la parte B, los grupos de edad de más de 50 años. De los 22 grupos de edad, en 13 se observó predominio de las mujeres. La mayor diferencia se encontró en el grupo de 30 a 34 años del departamento de Chocó (^4^,^5^) y, la menor, en el de 21 a 24 años en el departamento de Nariño (0,01) y el de 90 a 94 años en el departamento del Valle (0,01). Se evidenció que, en la primera etapa de la vida, es decir, entre los grupos de edad menores de un año y hasta los 4 años, hubo predominio de las mujeres. Posteriormente, durante la niñez tardía y la adolescencia, el predominio femenino descendió y se centró en los hombres. Más adelante, se apreció nuevamente un mayor reporte en las mujeres del grupo de 21 a 24 años en los cuatro departamentos, tendencia que permaneció hasta el grupo de 35 a 39 años y después descendió nuevamente, evidenciando predominio de los hombres. Otro pico en las mujeres se observó en el grupo de 50 a 54 años, patrón que se mantuvo hasta el grupo de 80 a 84 años.

**Cuadro 1:** Diferencia absoluta por región y grupo etario

Edad (años)	Cauca	Chocó	Nariño	Valle del Cauca
< 1	0,19	0,79	-0,09	0,38
1-4	0,35	0,69	-1,07	-0,01
5-9	-0,64	-2,17	-0,77	0,04
10-13	1,26	-0,86	-0,99	-0,57
14-16	-0,21	-0,55	-0,67	-0,03
17-20	-0,03	-2,34	-0,37	-0,76
21-24	0,81	0,10	0,01	0,38
25-29	-3,33	-3,24	1,63	1,93
30-34	-0,33	4,51	2,04	-0,49
35-39	-2,40	0,10	0,77	0,76
40-44	-0,96	-1,69	-0,24	-0,74
45-49	-1,37	0,98	-2,36	-0,27
50-54	3,40	3,96	1,68	0,51
55-59	1,18	0,31	0,08	-0,70
60-64	1,34	-0,60	-0,67	-0,88
65-69	-0,25	-2,55	0,67	-0,55
70-74	1,30	0,60	-1,58	-0,07
75-79	0,78	1,91	1,76	0,17
80-84	-0,03	0,45	0,82	0,25
85-89	-0,23	-0,88	-0,34	0,42
90-94	-0,84	0,48	-0,35	0,01
95-99	0,00	0,00	0,03	0,21

La frecuencia de los grupos de edad con predominio de mujeres mayores de 50 años, se observó en todos los departamentos, por lo que los siguientes análisis se enfocaron en este grupo de edad. La mayor diferencia se encontró en el grupo de 50 a 54 años en el departamento de Chocó (^3^,^9^) y, la menor, en el de 90 a 94 años en el departamento de Nariño (0,03). La diferencia de mayor magnitud en todos los departamentos se detectó en el grupo de 50 a 54 años y, a partir de allí, fue descendiendo progresivamente hasta el grupo de 95 a 99 años, con la única excepción del grupo de edad entre los 60 y 69 años, en el cual se reportó predominio masculino. El número de grupos de edad con predominio de las mujeres fue similar en todos los departamentos, excepto en Cauca, donde la diferencia estuvo principalmente en los grupos de los adultos mayores con menor edad, así como en el departamento del Valle, donde lo estuvo entre los adultos mayores de edad más avanzada. El pico entre los 50 y los 54 años se observó de manera persistente en los cuatro departamentos.

Para completar la descripción del nivel departamental, se presentan las tablas en las que se agrupan los municipios según la proporción de población concentrada en la cabecera municipal (cuadro 2). Allí se observa la variación de un departamento a otro en cuanto a los rangos de edad en que las mujeres fueron la población en la que más se reportó este diagnóstico en comparación con los hombres. En el departamento del Cauca, el comportamiento de los municipios concentrados fue similar al de los dispersos, siendo más parecido en el rango de edad de 50 a 64 años, con un mayor riesgo de reporte de este diagnóstico entre las mujeres. En cuanto al Chocó, llamó la atención que la población femenina con este diagnóstico pertenecía principalmente a los grupos de jóvenes, en los municipios concentrados; sin embargo, en los municipios dispersos, las mayores diferencias se observaron en la población adulta mayor, llegando incluso hasta los 84 años. En el caso de Nariño, hubo intermitencia del predominio femenino en los grupos de edad, tanto en los municipios concentrados como en los dispersos, con una leve tendencia hacia los adultos mayores en los territorios rurales. Por último, en el departamento del Valle, se evidenció que en los municipios concentrados se reportó más el diagnóstico B24 en los extremos de la vida, en tanto que, en los municipios dispersos, se observó una concentración en los adultos jóvenes.

**Cuadro 2:** Distribución por municipios (disperso Vs. concentrado) en los cuatro departamentos estudiados, y diferencias mínimas y máximas según grupo etario

Departamento	Cauca	Choco	Nariño	Valle
Número de municipios	42	27	62	41
Municipios dispersos n (%)	39 (92,8 %)	25 (92,5 %)	60 (96,7 %)	30 (73,1 %)
**Municipios dispersos**				
Número de grupos etarios con predominio femenino				
0-14 años (5)	2/5	2/5	0	3/5
15-49 años (8)	3/8	2/8	3/8	5/8
≥50 años (9)	6/9	6/9	6/9	2/9
Diferencias absolutas por grupo etario (min-max)				
0-14 años (5)	(0,35-1,63)	(1,43)	0	(0,17-1,29)
15-49 años (8)	(0,04-0,92)	(2,39-7,28)	(0,95-2,11)	(0,19-2,84)
≥50 años (9)	(0,30-4,35)	(1,28-6,17)	(0,09-2,25)	(0,10-0,60)
**Municipios concentrados**				
Número de grupos etarios con predominio femenino				
0-14 años (5)	3/5	2/5	2/5	2/5
15-49 años (8)	4 (50 %)	4 (50 %)	4 (50 %)	3 (37,5 %)
≥50 años (9)	4 (44,4 %)	2 (25 %)	2 (25 %)	5 (55,5 %)
Diferencias absolutas por grupo etario (min-max)				
0-14 años (5)	(0,17-1,04)	(0,33-0,58)	(0,24-0,50)	(0,00-0,42)
15-49 años (8)	(0,19-1,51)	(0,50-5,63)	(1,08-2,61)	(0,41-2,05)
≥50 años (9)	(1,04-2,64)	(0,71)	(0,28-1,80)	(0,17-0,49)

## Discusión

Los resultados indican que las diferencias observadas se pueden agrupar en tres segmentos del ciclo vital: al inicio de la vida, en que predominó el reporte en las mujeres; en la adolescencia y la etapa de adulto joven, con predominio de reporte en los hombres, y, por último, entre los adultos mayores, en quienes nuevamente predominó el reporte de mujeres.

El hallazgo más relevante en los cuatro departamentos estudiados señala que los valores más elevados de reporte en mujeres ocurrieron entre las edades de 21 a 24 y de 50 a 54 años, y que las proporciones en las edades de 50 a 54 años fueron mayores que en el primer grupo. Esto podría explicarse por el efecto de la cohorte de nacimiento, pues el grupo de mujeres mayores de 50 años tenía alrededor de 20 años en la década de los 80, momento histórico en el que aún no se habían constituido muchos de los programas de prevención y promoción que hoy están disponibles.

El análisis de las diferencias por grupos de edad permitió establecer dos comportamientos distintos: antes de los 50 años de edad, cuando el predominio y la concentración de reportes fue para los hombres, y después de los 50 años de edad, cuando las diferencias mostraron predominio de las mujeres. Esto podría corresponder al efecto poblacional que tiene la “generación X” en la presentación de la condición. Las personas nacidas entre 1965 y 1980, y las generaciones previas, muestran un comportamiento diferente a las cohortes de nacimiento posteriores a 1980. Además, hay que tener en cuenta que la terapia antirretroviral solo comenzó a emplearse en los años 90.

Al efecto de la cohorte de nacimiento se suma un componente estructural de nivel contextual que se relaciona con los servicios de salud. En el análisis también se evidenció la variabilidad por departamentos, con diferencias de mayor magnitud en Cauca y Chocó, entes territoriales con menor nivel organizativo y experiencia que el Valle del Cauca y Nariño, en cuanto al grado de consolidación de los programas de atención de HIV.

En Colombia, los mayores esfuerzos de prevención del HIV se han concentrado en los adolescentes, grupo de edad en que se inicia la vida sexual y se tiene mayor riesgo de transmisión. Sin embargo, los resultados de este análisis demuestran que es necesario incluir también en la agenda a los grupos de adultos mayores. Esta recomendación es coherente con el análisis de las tendencias mundiales y regionales entre personas de más de 50 años con HIV en el periodo de 2000 a 2020 (^10^), así como con los datos de encuestas poblacionales (^11^) y de incidencia obtenidos a partir de la vigilancia en salud pública (^12^).

El patrón observado es similar al reportado en otros países; por ejemplo, en Estados Unidos el 10 % de los casos nuevos de sida ocurre en personas mayores de 60 años y, en el 2015, más de la mitad de la población diagnosticada con HIV era mayor de 50 años (^13^). Según los CDC, en el 2018, el 17 % de los diagnósticos de nuevos casos de HIV en ese país se presentó en población mayor de 50 años y de estos, el 43 % se encontraba entre 50 y 54 años (^2^). En Europa Occidental, el 10 % de los casos nuevos ocurre en el grupo de personas mayores de 50 años, con 4,3 % en Europa Central y 0,7 % en Europa Oriental.

En España, casi el 7 % de los casos registrados se presenta en mayores de 50 años (^14^). En un estudio del 2004 en este país, se analizaron las características epidemiológicas, clínicas y evolutivas de una serie clínica de pacientes mayores de 50 años infectados con el HIV en el momento del diagnóstico. Se encontró que había habido un aumento de casos de infección en personas mayores de 50 años, y que el mecanismo de transmisión más frecuente era la vía sexual. De los 165 pacientes incluidos en el estudio, el promedio de edad en el momento del diagnóstico fue de 58,4 años y el 81 % había adquirido la infección por la vía sexual; la mayoría de los diagnósticos se hicieron ante la aparición de una enfermedad oportunista, y la mortalidad se situó en el 32,7 % (^15^). Además, en el documento de consenso sobre edad avanzada e infección por el HIV publicado en el 2015 por la Sociedad Española de Geriatría y Gerontología, se demostró que hay un número creciente de personas mayores de 50 años con HIV en el mundo actual, sobre todo en los países de ingresos bajos y medios donde hay pocas estrategias que atiendan esta fase de la epidemia (^16^).

Este pico de prevalencia poblacional puede deberse en gran medida a la disminución de la incidencia del HIV entre los adultos más jóvenes, con lo que la carga de morbilidad se desplaza a edades más avanzadas, y a la baja percepción del riesgo de la infección a estas edades, lo que hace que las personas mayores de 50 años practiquen conductas de riesgo, como no usar preservativos por considerarlos un método anticonceptivo y no de prevención de las infecciones de transmisión sexual. Además, la cultura machista que aún prevalece normaliza que los hombres, incluso a esa edad, mantengan relaciones sexuales por fuera de la relación de pareja, en tanto que para las mujeres mayores la sexualidad sigue siendo un tema tabú en los servicios sociales, la sociedad y entre ellas mismas, lo que las condiciona a creer que el sexo es una actividad inconveniente a partir de cierta edad (^17^). Por último, pero no menos importante, los cambios biológicos tienen un papel importante, pues después de la menopausia la pared vaginal se vuelve más delgada, con lo que aumentan las posibilidades de sufrir lesiones y desgarros, los cuales pueden ser la vía de entrada del virus durante las relaciones sexuales (^16^). Por otro lado, el uso de antirretrovirales aumenta las tasas de supervivencia en la población con HIV, lo que se traduce en una mayor prevalencia en la población mayor. Es el caso de España, donde se ha evidenciado que la población con HIV está envejeciendo gracias a la terapia antirretroviral, lo que aumenta tanto la supervivencia como los nuevos casos entre los adultos mayores, la mitad de los cuales viven con infección por HIV, en tanto que alrededor del 20 % de los nuevos casos corresponde a personas mayores de 50 años (^18^).

Es injustificable que no haya esfuerzos para generar programas orientados a este grupo poblacional, lo que puede deberse a que los signos de la enfermedad en estas edades se pueden confundir con los malestares y dolores del envejecimiento normal. Cada vez hay mayor evidencia en la literatura científica que sugiere que la población con infección por HIV experimenta cambios inmunológicos similares a los que provoca el envejecimiento en la población anciana sin infección (^16^). Por otro lado, las personas mayores tienen menos probabilidad de hacerse la prueba, ya que pueden sentir vergüenza o temor por el resultado, incluso muchos profesionales en medicina no consideran la tamización para HIV en este grupo poblacional (^19^).

Otra posible explicación es que los planificadores de políticas y servicios pueden considerar, erróneamente, que las personas de esa edad no tuvieron ni tienen relaciones sexuales, por lo tanto, no se invierte en estudios o programas dirigidos especialmente a este grupo de alto riesgo. En 2006, el artículo titulado “El sida y las personas mayores” demostró que, para ese momento, la bibliografía internacional sobre HIV se concentraba principalmente en las poblaciones de 14 a 49 años, y señaló las características particulares por las cuales se requieren estudios orientados a la población adulta mayor: muchas personas de esta edad vuelven a estar solteras (divorciadas o viudas) y en sus relaciones de pareja no prestan atención a los mensajes de prevención; los investigadores y los servicios de salud no reconocen las necesidades sexuales de los mayores; además, existen barreras sociales para dialogar sobre su sexualidad y los profesionales de la salud no les preguntan sobre su orientación sexual o sus comportamientos sexuales de riesgo (^14^).

Entre las limitaciones del estudio pueden mencionarse los sesgos de selección, pues si bien la notificación al SISPRO es obligatoria, la oportunidad y exhaustividad de su diligenciamiento es diferente en los territorios urbanos y los rurales. Asimismo, podría considerarse la variabilidad interanual asociada al manejo de la plataforma, aunque se presume que para el 2014, primer año del periodo de estudio, los prestadores de servicios ya tenían cinco años de experiencia con el sistema.

Para concluir, el análisis secundario de una base de datos de recolección rutinaria de información es útil, especialmente cuando se dispone de un gran número de eventos, ya que, si el enfoque analítico es coherente, se pueden detectar patrones específicos. Este tipo de estudios posibilita el análisis en escalas territoriales subnacionales que habitualmente no se consideran en la investigación y la planeación de intervenciones en salud pública.

Para poder realizar un seguimiento adecuado y periódico de esta enfermedad, el código CIE-10 de mayor utilidad en Colombia es el B24 “Enfermedad por VIH”, ya que es el que los profesionales de salud más usan para la notificación.

Los datos analizados evidencian una representación poblacional significativa de adultos mayores, especialmente mujeres, entre las personas que consultaron por HIV en el periodo de estudio, por lo que es importante considerar la inclusión de estrategias de promoción y prevención acordes con las necesidades y características particulares de la población mayor de 50 años en el programa nacional de HIV.
